# Human Hypertension Blood Flow Model Using Fractional Calculus

**DOI:** 10.3389/fphys.2022.838593

**Published:** 2022-03-22

**Authors:** Mohamed A. Bahloul, Yasser Aboelkassem, Taous-Meriem Laleg-Kirati

**Affiliations:** ^1^Computer, Electrical, and Mathematical Sciences, and Engineering Division (CEMSE), King Abdullah University of Science and Technology (KAUST), Thuwal, Saudi Arabia; ^2^College of Innovation and Technology, University of Michigan, Flint, MI, United States; ^3^Michigan Institute for Data Science, University of Michigan, Ann Arbor, MI, United States; ^4^National Institute for Research in Digital Science and Technology (INRIA), Paris, France

**Keywords:** blood flow, hypertension, vascular compliance, fractional calculus, Windkessel model

## Abstract

The blood flow dynamics in human arteries with hypertension disease is modeled using fractional calculus. The mathematical model is constructed using five-element lumped parameter arterial Windkessel representation. Fractional-order capacitors are used to represent the elastic properties of both proximal large arteries and distal small arteries measured from the heart aortic root. The proposed fractional model offers high flexibility in characterizing the arterial complex tree network. The results illustrate the validity of the new model and the physiological interpretability of the fractional differentiation order through a set of validation using human hypertensive patients. In addition, the results show that the fractional-order modeling approach yield a great potential to improve the understanding of the structural and functional changes in the large and small arteries due to hypertension disease.

## 1. Introduction

Cardiovascular diseases (CVDs) are the leading cause of death worldwide, responsible for more than 17.9 million deaths in 2019 that representing 32% of global mortality. This number is expected to reach 23.6 million by 2030 (Mensah et al., 2019). A key risk factor for CVDs is high blood pressure, known as hypertension. Although the reduction in hypertension can restrain the onset of CVDs, current treatments techniques are only partially effective. In fact, hypertension is considered chronic pathology that can only be regulated with medication; however, it cannot be cured definitely. The primary pathological sign of high blood pressure is reduced vascular compliance due to structural remodeling and functional modifications in the arteries. In a normotensive state, any variation of the hemodynamic induces structural and functional adaptations within the different cell types and layers of the vascular wall. However, in hypertensive states, this adaptive response does not lead to normal hemodynamic control but instead inducts irregular vascular changes, defined as, vascular remodeling (Brown et al., 2018). Several clinical studies in-patient and experimental researches have revealed the marked correlation between vascular remodeling and the pathophysiology of hypertension. In particular, they observe that the vascular remodeling in resistive arteries is firmly associated with the progression and severity of hypertension's disease. Accordingly, deep understanding and analysis of the pathological mechanisms of hypertension vascular remodeling hold high significance for diagnosing CVDs and is crucial for the clinical treatment of hypertension (Li et al., 2017).

Over the last century, various physics-driven and data-driven modeling methods and diverse numerical computational approaches have been developed to characterize vascular biomechanics and arterial hemodynamics. Commonly, these approaches involve a compromise between precision and complexity. In the open literature, the arterial hemodynamics and mechanic modeling approaches are classified into two main classes: macro and micro modeling methods. The macro-scale class is considered a low dimensional strategy that usually implicates the well-known lumped parametric, the arterial Windkessel, (Frank, 1899). Typically this class used ordinary differential equations (ODEs) to describe the arterial hemodynamic as a function of time only. Accordingly, they are commonly explored to describe the global cardiovascular functions and biomechanical properties (Shi et al., 2011; Malatos et al., 2016). This class is considered computationally simple but less accurate than the microscale models. In addition, it is less insightful in terms of physiological interpretability, (Zhou et al., 2019). On the other hand, the microscale models are generally considered more insightful as they provide a precise estimate of cardiovascular function and accurately represent local as well as global arterial biomechanical properties. These modeling approaches are considered high-dimensional paradigms [one-dimensional (1D), two-dimensional (2D), and three-dimensional (3D)] as they involve more than one dimension along with the time scale to describe the complex geometries of the arterial network. Although micro-scale-based models provide detailed information about the arterial circulatory system, their complexity is not manageable in practical medical routines, (Zhou et al., 2019).

In recent decades, fractional-order models have surfaced as potential techniques that compromise between accuracy and computational cost for large-scale problems in different fields (Bahloul and Kirati, 2021). In particular fractional-order differential equations have been considerably explored in modeling complex biological systems (Magin, 2006). Basically, fractional-order approaches allow much modeling flexibility by extending the concepts of differentiability and integrating the non-local and memory properties through the fractional differentiation order. These features enable the characterization of complex phenomena over various time and space scales without splitting the problem into smaller sub-compartments.

The versatility and flexibility of fractional-order tools lead researchers to believe that the future of computational modeling in bio-engineering and bio-informatics (Magin, 2006). This paradigm shift extends from bio-engineering in general to cardiovascular system modeling and characterization specifically, as experimental studies find that fractional-order models are more suitable and interpretable in describing the arterial structure and biomechanical response of the heart and systemic circulation. *In-vivo* and *in-vitro* investigations have pointed that fractional-order calculus-based approaches are more convenient to precisely represent the viscoelasticity properties of soft collagenous tissues in the vascular bed. A fractional-order viscoelastic model in human arterial segments has been tested and validated in (Craiem et al., 2008). Results show that the extra fractional-differentiation order parameter (α) allowed predicting complex and frequency-dependent responses similar to reported complex elastic moduli in arteries. (Craiem and Armentano, 2007; Craiem et al., 2008; Zerpa et al., 2015) revealed that fractional models should be acknowledged adequate alternatives to model arterial viscoelasticity. In addition, the fractional differentiation order (α) plays a pivotal function, and it is considered the most valuable parameter to monitor and analyze, reflecting the structural and functional changes in the arteries. Most recently, we have used fractional-order derivative to model the apparent arterial compliance dynamics. The proposed model employs fractional-order capacitor (FOC) element that combine the complex and frequency dependence characteristics of arterial compliance. The FOC modeling approach accounted for both resistive and capacitive properties allowing a reduced-order representation of the vascular compliance and stiffness (Bahloul and Kirati, 2021).

**Figure 1:**
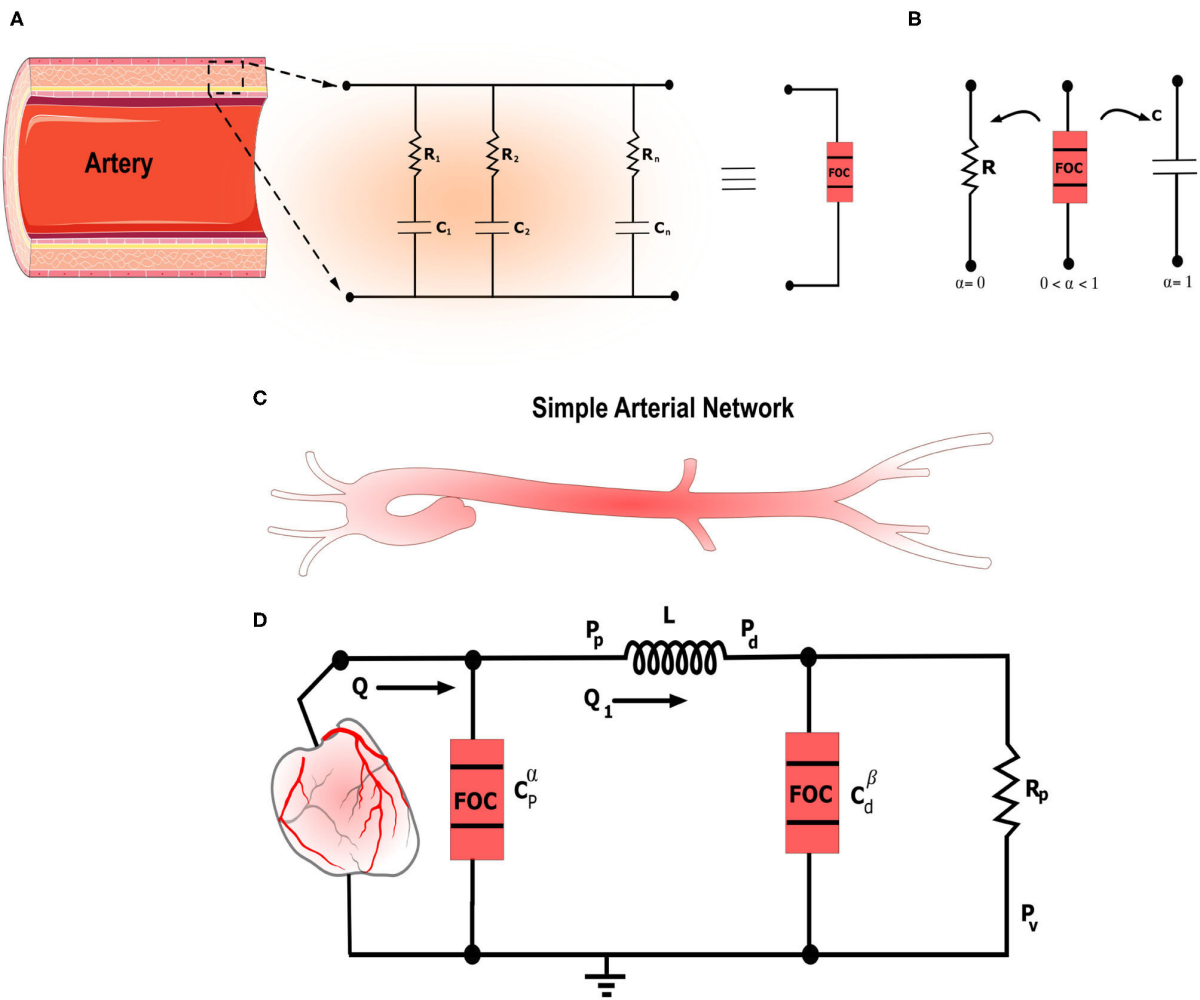
The figure illustrates the general framework for fractional-order modeling of the arterial system in a schematic manner. **(A)** Schematic illustrating the artery along with the equivalent *RC* tree equivalent circuit of the fractional-order capacitor. **(B)** Electrical symbols of the standard resistor and capacitor and the fractional-order capacitor. 0 < α <1 represents the fractional differentiation order. For α = 0 and α = 1 the fractional-order capacitor is equivalent to the standard resistor and capacitor, respectively. **(C)** Schematic that shows simplified left ventricle-aortic-arteries sub-domains. **(D)** Electrical analog of the proposed fractional-order modified Windkessel (F-MWK) model.

This study presents a novel fractional-order modified Windkessel model to study the blood flow in the arterial system. Fractional-order tools have been adopted to represent the dynamic relationship between blood pressure and volume in the proximal and distal sites of the arterial network. Accordingly, the model includes two fractional-order capacitors to describe the apparent vascular compliance of large arteries close to the heart and one of the resistive arteries further away from the heart as illustrated in Figure 1C. The proposed model has been applied and validated using two hypertension datasets acquired from human subjects. The results show the accuracy and flexibility of the proposed method in modeling the aortic blood pressure measurements while maintaining a low model complexity. In addition, the proposed fractional-order representation draws a more appropriate framework to analyze and understand hypertension behavior and its pathophysiology connection with vascular remodeling.

## 2. Materials and Methods

### 2.1. Fractional-Order Calculus

In the last decades, non-integer differentiation, the so-called fractional-order calculus, became a popular tool for characterizing real-world physical systems and complex behaviors from various fields such as biology, control, electronics, and economics (Gutiérrez et al., 2010; Magin, 2010). The long-memory and spatial dependence phenomena inherent to the fractional-order systems present unique and attractive peculiarities that raise exciting opportunities to represent complex phenomena subject to power-law behavior accurately. For instance, the power-law behavior has been demonstrated in describing human soft tissues visco-elasticity and characterizing the elastic vascular arteries. *In-vivo* and *in-vitro* experimental studies have pointed that fractional-order calculus-based approaches are more decent to precisely represent the hemodynamic; the viscoelasticity properties of soft collagenous tissues in the vascular bed; the aortic blood dynamics (Perdikaris and Karniadakis, 2014; Zerpa et al., 2015); red blood cell (RBC) membrane mechanical properties (Craiem and Magin, 2010); and the heart valve cusp (Doehring et al., 2005; Craiem and Armentano, 2007; Craiem et al., 2008; Zerpa et al., 2015).

The continuous fractional integro-differential operator $D_{t}^{\alpha }$ is defined as follows


(1) $$D_{t}^{\alpha }=\left\{ \begin{array}{l} \frac{d^{\alpha }}{dt^{\alpha }},\;\alpha >0,\;\\ 1,\;\alpha =0\\ \int_{a}^{t}{\left(d\tau \right)}^{\alpha },\;\alpha <0 \end{array}\right.$$

where α is the fractional differential integral order.

Several definitions for fractional-order derivative exist in the literature (Podlubny, 1999), (Lorenzo and Hartley, 1998). In this work, we consider the *Grunwald-Letnikov (GL)* definition given as:

***Definition 1**. (Podlubny, 1999) The Grunwald-Letnikov derivative of order α of a function *f*, denoted $D_{t}^{\alpha }f\left(t\right)$, is given by:*


(2) $$\begin{array}{r} D_{t}^{\alpha }f\left(t\right)=\underset{h\to 0}{\lim}\frac{1}{h^{\alpha }}\overset{\infty }{\underset{i=0}{\sum }}c_{i}^{\left(\alpha \right)}f\left(t-ih\right),\alpha >0, \end{array}$$

*where *h*>0 is the time step, $c_{i}^{\left(\alpha \right)}\left(i=0,1,...\right)$ are the binomial coefficients recursively computed using the following formula,*


(3) $$\begin{array}{r} c_{0}^{\left(\alpha \right)}=1,\;c_{i}^{\left(\alpha \right)}=\left(1-\frac{1+\alpha }{i}\right)c_{i-1}^{\left(\alpha \right)}. \end{array}$$

### 2.2. Fractional-Order Capacitor

**Figure 2:**
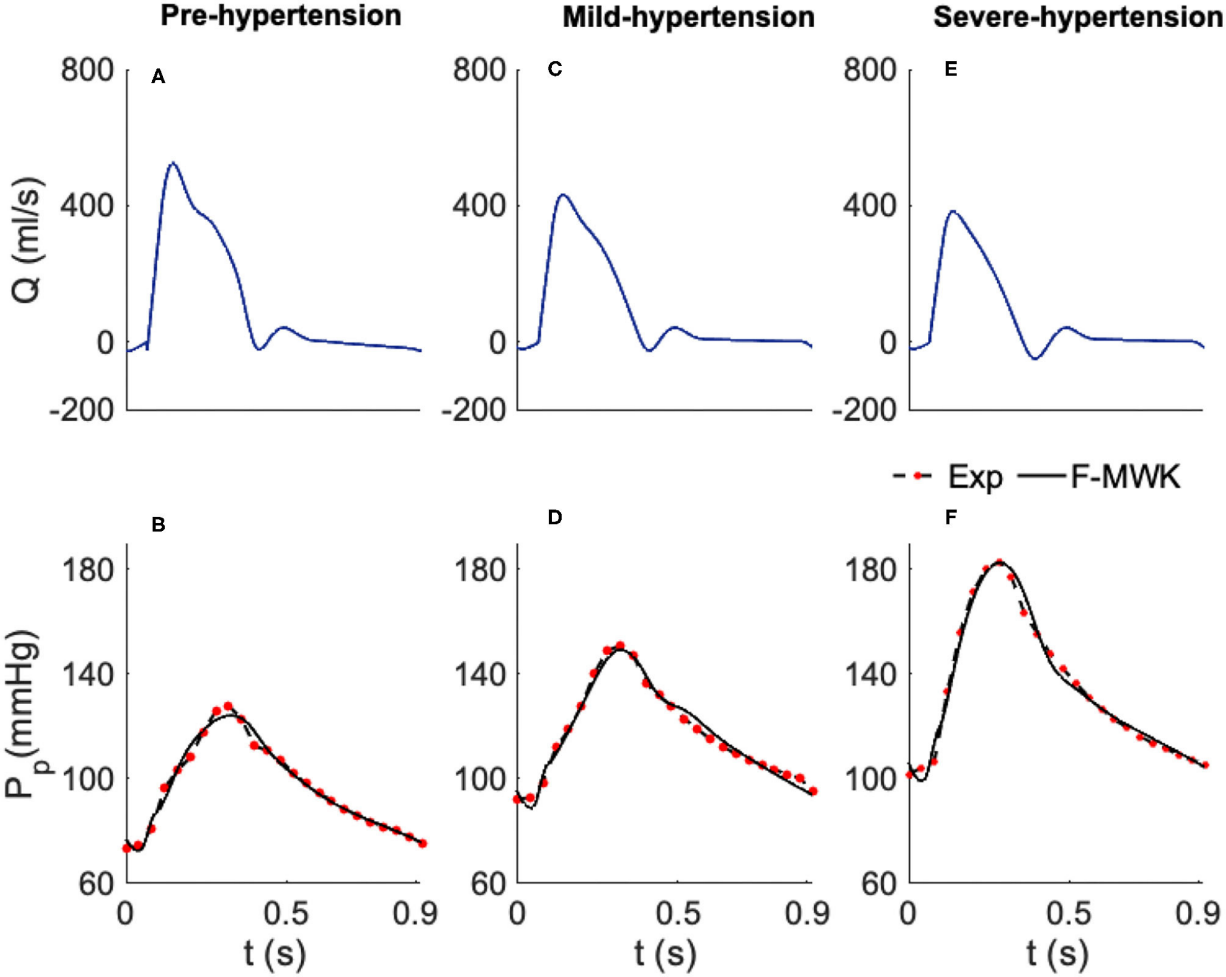
Estimated proximal blood pressure using the proposed fractional-order modified Windkessel model along with the experimental *in-vivo* human-hypertension. **(A,C,E)** Represent the model input aortic-valve blood flow rate for each validation case of study, Pre-hypertension, Mild-hypertension, and Sever-hypertension, respectively. **(B,D,F)** Represent the pressure waveform validation results.

Fractional-order capacitor (FOC) known as the constant phase element is a fractional-order electrical element representing the fractional-order derivative through its *curent-volatge* characteristic. In fact, the relationship between the current, *i*(*t*), passing through an FOC and the voltage, *v*(*t*), across it with respect to time, *t*, can be written as follow:


(4) $$\begin{array}{r} i\left(t\right)=C_{\alpha }\frac{d^{\alpha }}{dt^{\alpha }}v\left(t\right), \end{array}$$

where *C*_α_ is a proportionality constant so-called pseudo-capacitance, expressed in units of [Farad/second^1−α^], (Elwakil, 2010). The conventional capacitance, *C*, in unit of Farad is related to *C*_α_ as $C=C_{\alpha }\omega^{\alpha -1}$ that is frequency-dependent. The fractional-order impedance (*Z*_α_) is expressed as follow:


(5) $$\begin{array}{r} Z_{\alpha }\left(s\right)=\frac{1}{C_{\alpha }s^{\alpha }}=\underset{Z_{r}}{\underset{︸}{\frac{1}{C_{\alpha }}\omega^{-\alpha }\cos\left(\phi \right)}}-j\underset{Z_{i}}{\underset{︸}{\frac{1}{C_{\alpha }}\omega^{\alpha }\sin\left(\phi \right)}}, \end{array}$$

where *s* corresponds to the *Laplace* variable and ϕ denotes the phase shift expressed as: ϕ = απ/2 [rad] or ϕ = 90α [degree or °]. *Z*_*r*_ and *Z*_*i*_ are the real and imaginary parts of *Z*_α_ corresponding to the resistive and capacitive portions, respectively. From (5), it is apparent that the transition between resistive and capacitive parts is ensured by α. If 0 ≤ α ≤ 1, the bounding conditions of α will corresponds to the discrete conventional elements: the resistor at α = 0 and the ideal capacitor at α = 1, as illustrated in Figure 1B. As α goes to 0, (*Z*_*i*_) convergence to 0, and thus the fractional element looks like that a pure resistor, whereas as α goes to 1, (*Z*_*r*_) converges to 0 and hence, the fractional element serves as a pure capacitor, (Oustaloup et al., 2000; Krishna et al., 2011; Hartley et al., 2015; Trigeassou and Maamri, 2020). Figure 2B represents the schematic diagram for a FOC along with the ideal resistor and capacitor. Many studies have shown that FOC is equivalent to a resistor ladder network (RC tree circuit), (Carlson and Halijak, 1964; Si et al., 2017). This structure is similar to the electrical analogy of the generalized Kelvin-Voigt viscoelastic model. Figure 1A presents the equivalent RC tree circuit of FOC of any order. Bearing these properties in mind, the fractional differentiation order α parameter allows extra versatility in modeling viscoelastic systems (Vastarouchas and Psychalinos, 2017).

### 2.3. Apparent Arterial Compliance

Arterial compliance stands for the ability of the vessel to store the blood. Functionally, it is defined as the ratio of the incremental variation in the blood volume (*dV*) due to an incremental variation in distending pressure (*dP*). Accordingly, mathematically it is expressed as: *C* = *dV*/*dP*, (Quick et al., 1998). Over the last decades, several analytical and experimental studies have focused on modeling and characterizing vascular compliance, (Chemla et al., 1998; de Simone et al., 1999; Segers et al., 1999; Stergiopulos et al., 1999; Mackenzie et al., 2002; Westerhof et al., 2009; Haluska et al., 2010; Ge, 2018; Kaya et al., 2018). With the introduction of the well-known linear Windkessel representation of the arterial system, arterial compliance was assumed to have a single constant value for the entire cardiac cycle. Hence, the transfer function relating the blood volume variation to the blood pressure input changes was also considered constant. Accordingly, the arterial compliance was modeled within the arterial lumped parameter circuit's Windkessel as an ideal capacitor whose capacitance is constant (Westerhof et al., 2009). However, this assumption was not realistic, and its drawbacks were reflected essentially in the estimation of the hemodynamic determinants (Quick et al., 2000). In fact, it does not lead to the correct evaluation of the true value of arterial compliance (Craiem and Armentano, 2003). Besides, by analyzing the transfer function blood volume/input pressure, experimental studies have shown that this relationship is frequency-dependent, and a time delay between the arterial blood volume and the input blood pressure is observed. Hence a variation in the arterial compliance along the cardiac cycle coexists (Burattini and Natalucci, 1998; Quick et al., 1998, 2000).

In order to take into account this frequency dependence, some research investigations have promoted a new configuration where they considered the viscoelastic properties of the arterial vessel and represented the arterial compliance using the so-called *Voigt-cell* configuration (Burattini and Natalucci, 1998; Aboelkassem and Virag, 2019). This type of arterial model was known as viscoelastic Windkessel. Although the viscoelastic *Voigt-cell* has resolved some contradictions of the standard elastic lumped parameter Windkessel, this configuration does also present some limitations as it does not account for the so-called stress-relaxation experiment (Burattini and Natalucci, 1998). To overcome this restriction, high-order viscoelastic configurations have been proposed by connecting many Voigt-cells as shown in Figure 1A. This solution might lead to an accurate estimation of arterial compliance and its feature; however, it is deemed a very complex alternative that poses extra challenges. Indeed, the number of parameters to estimate is more significant for higher-order models, while the obtained experimental data is habitually small and insufficient to identify all the parameters. It is also known that reduced-order models are more desirable for their uniformity and simplicity of investigation (Burattini and Natalucci, 1998; Bahloul and Kirati, 2019).

Bearing this in mind, recently, an alternative modeling approach of the apparent compliance was proposed in (Bahloul and Kirati, 2021) where fractional-order tools were investigated to represent the complex phenomena underlying the apparent arterial compliance. In (Bahloul and Kirati, 2021), the authors presented fractional-order models to describe the dynamic relationship between aortic blood pressure and volume, describing the apparent vascular compliance. The proposed model employs fractional-order capacitor elements to lump the complex and frequency dependence characteristics of arterial compliance. FOC combines both resistive and capacitive properties, which the fractional differentiation order, α, can control. The validations results find that the fractional-order scheme can reconstruct the overall dynamic of the complex and frequency-dependent apparent compliance dynamic and reduce the complexity.

The vascular apparent compliance in the fractional-order domain is defined as follows:


(6) $$\begin{array}{r} Q_{stored}\left(t\right)=\frac{d^{\alpha }V}{dt^{\alpha }}=\underset{C_{app}^{\alpha }}{\underset{︸}{\frac{d^{\alpha }V\left(t\right)}{d^{\alpha }P_{in}\left(t\right)}}}\frac{d^{\alpha }P_{in}\left(t\right)}{dt^{\alpha }}, \end{array}$$

where *Q*_*stored*_ is the blood stored in the arterial tree, *V* corresponds to the blood volume, and *P*_*in*_ is the input blood pressure.

The FOC can be an inherent lumped element that can catch vascular compliance's complex and frequency-dependent behavior. In fact, as expressed in (6), the pseudo compliance, *C*_α_*app*__, should be expressed in the unit of [ml/mmHg.sec^1−α^] that makes, naturally, the standard compliance (*C*_*C*_), in the unit of [ml/mmHg], frequency-dependent as:


(7) $$\begin{array}{r} C_{C}=C_{app}^{\alpha }{\left(j\omega \right)}^{\alpha -1}. \end{array}$$

Hence, the fractional-order capacitor presents physical bases in portraying the complex and frequency dependency of the apparent vascular compliance. Besides, based on the variation of the fractional differentiation order α, the real and imaginary parts of the resultant FOC's impedance can possess various levels, so by analogy, α can control dissipative and storage mechanisms and hence the viscous and elastic component of the arterial wall. Furthermore, it is worth remarking that the equivalent circuit representation of FOC can be seen as an infinity Voigt cells branches joined in parallel. Consequently, FOC simplifies the representation of the complex arterial network's mechanical properties by employing only two parameters (α and *C*_α_).

### 2.4. Fractional-Order Modified Windkessel Model

The modified Windkessel model (MWK) is one of the simplest arterial representation that lumps the arterial network into two main compartments, proximal and distal, (Goldwyn and Watt, 1967). Taking into account that the proximal arteries close to the heart have different properties in comparison to the distal ones, MWK splits the total arterial compliance used in the original arterial Windkessel into two capacitances: *C*_*p*_ represents the compliance of the large arteries which are commonly elastic and *C*_*d*_ depicts the compliance of muscular arteries that are more stiffer. Clinical studies demonstrated that *C*_*d*_ is very sensitive to vasodilatory experiments, a property apparent in distal arteries. Other investigations have also shown that *C*_*d*_ is reduced with aging and hypertension. The latest properties make these capacitance as potential indicators of cardiovascular risk, (Francis, 2007). MWK comprises an inductor, *L*, between the two capacitance accounting for the inertance of the flowing blood. Also, it lumps the peripheral resistance into a resistor *R*_*p*_ and the venous pressure into a constant *P*_*v*_. Based on the electrical analogy, the pumping heart is modeled as a pulsating current source.

In this study, we propose a general version of the MWK using fractional-order framework. We present the fractional-order modified Windkessel (F-MWK) representation as shown in Figure 1D. The proposed model comprises two fractional-order capacitor $C_{p}^{\alpha }$ and $C_{d}^{\beta }$ to take into account the apparent vascular compliance of the large and small arteries, respectively. Figure 1D shows the schematic of F-MWK. As the proposed model contains two fractional-order storage elements and one integer-order one, three states are needed to describe the dynamic of the system. The aortic blood flow *Q*(*t*) ejected from the left ventricle is considered the input to the system. *P*_*p*_ denotes the aortic proximal pressure and *P*_*d*_ represents the distal blood pressure. *Q*_1_ represents the blood flow throughout the inertia *L*. Applying the Kirchhoff's voltage and current laws to the circuit shown in Figure 1D. We obtain the following equations:


(8) $$\left\{ \begin{array}{l} C_{p}^{\alpha }\cdot \frac{d^{\alpha }}{dt^{\alpha }}P_{p}(t)+Q_{1}(t)=Q(t)\\ \frac{P_{p}(t)}{L}-\frac{P_{d}(t)}{L}=\frac{dQ_{1}(t)}{dt}\\ C_{d}^{\beta }\cdot \frac{d^{\beta }}{dt^{\beta }}P_{d}(t)+\frac{P_{d}(t)-P_{v}}{R_{p}}=Q_{1}(t) \end{array}\right.$$

The resulting pseudo-state space representation is then:


(9) $$\begin{array}{r} D_{t}^{q}x\left(t\right)=Ax\left(t\right)+B\left(x\right), \end{array}$$

where $D_{t}^{q}={\left[D_{t}^{\alpha },D_{t}^{\gamma },D_{t}^{\beta }\right]}^{tr}$ is the fractional-order derivative operator for all the states. (·)^*tr*^ denotes the transpose of the row vector. In this study γ = 1 as we used an integer-order inductor to connect the proximal and distal compartments. $xx={\left[P_{p},Q_{1},P_{d}\right]}^{tr}$ denotes the pseudo-states vector representing the aortic proximal, the blood flow throughout the inductor, and the distal pressure, respectively. The matrix *A* representing the lumped parameters is expressed as:


(10) $$\begin{array}{r} A=\left[\begin{array}{ccc} 0 & -\frac{1}{C_{p}^{\alpha }} & 0\\ \frac{1}{L} & 0 & -\frac{1}{L}\\ 0 & \frac{1}{C_{d}^{\beta }} & -\frac{1}{R_{p}C_{d}^{\beta }} \end{array}\right] \end{array}$$

*B* is written as:


(11) $$\begin{array}{r} {\left[\frac{Q\left(t\right)}{C_{p}^{\alpha }}\;0\;\frac{P_{d}\left(t\right)-P_{v}}{R_{p}}\right]}^{tr} \end{array}$$

***Remark 1**. If we were to assume that the pressure's drop across the blood inductor, *L*, is negligible, in this case, the proximal pressure is equal to the distal one. In addition, the F-MWK reduces to the simplest fractional-order two-element Windkessel model as proposed in (Bahloul and Laleg-Kirati, 2020) with an equivalent arterial compliance $C_{a}^{q}=C_{p}^{\alpha }+C_{d}^{\beta }$.*

## 3. Data and Analysis

### 3.1. *In-vivo* Human Hypertension Datasets

The proposed model was applied and validated using two real clinical datasets for human hypertension (Nichols et al., 1993; Li et al., 2017). The first *in-vivo* dataset (*n* = 3) was extracted and digitized from aging, and hypertensive studies (Nichols et al., 1993; Aboelkassem and Virag, 2019)). The data consists of measured aortic blood flow rate and aortic blood pressure for three human subjects suffering from three different hypertension stages, particularly Pre-hypertension, mild-hypertension, and severe hypertension. Their cardiac cycle is *T* = 0.92 *s*. The second clinical dataset was obtained from data supplement publicly available at in (Mariscal-Harana et al., 2021). The dataset was originally used in a study about forward and backward pressure waveform morphology analysis in hypertension, (Li et al., 2017). The dataset consists of 158 subjects assessed for hypertension (*n* = 158, 81 male, aged 46±17 years, mean ± SD). Based on (Li et al., 2017) the patients were recruited from those who were diagnosed with hypertension at Guy's and St Thomas' Hypertension Clinic. 48% of the subject were on treatment and hence their blood pressure were settled to the normal values (normotensive). More details about the collection of this data such as the consent approval and the measurement tools can be fount in (Mariscal-Harana et al., 2021). In addition to the aortic waveforms the dataset contains extra chracteristic of the subjects such as the height, weight, measurements of systolic (SBP) and diastolic (DBP) blood pressure, cardiac output, stroke volume and heart rate. As in this study we focus on the investigation of fractional-order framework for the assessment of hypertension, we divided the patients of this dataset into 4 groups corresponding to the range of SBP and DBP of the central blood pressure pulse: Group 1, normotension (DBP <80 AND SBP <120); Group 2, pre-hypertesion (80 ≤ DBP ≤ 85 OR 120 ≤ SBP ≤ 130); Group 3, hypertension (85 ≤ DBP ≤ 90 OR 130 < SBP ≤ 140) and Group 4, severe-hypertension (DBP>90 OR SBP>140). The characteristics of each group are listed in Table 1.

**Table 1:** Characteristics of the hypertensive clinical dataset 2.

	**Pulse pressure groups**			
**Subjects**	**Group 1**	**Group 2**	**Group 3**	**Group 4**
	**(*n* = 55)**	**(*n* = 41)**	**(*n* = 36)**	**(*n* = 26)**
Sex, male, [%]	45.45	58.53	50	50
	DBP <80	80 ≤ DBP ≤ 85	85 ≤ DBP ≤ 90	DBP>90
Characteristic	AND	OR	OR	OR
	SBP <120	120 ≤ SBP ≤ 130	130 < SBP ≤ 140	SBP>140
Age (years)	45.34 ±16.7	43.63 ±17.52	47.88 ±17.33	49.92 ±14.28
Height (m)	1.68 ±0.10	1.72 ±0.09	1.69 ±0.07	1.68 ±0.07
Weight (Kg)	74.76 ±15.68	79.69 ±13.75	77.81 ±14.50	80.18 ±17.16
DBP (mmHg)	70.40 ±6.24	82.65 ±7.03	88.36 ±7.29	95.53 ±15.76
SBP (mmHg)	105.23 ±10.10	124.88 ±8.02	136.71 ±11.52	159.03 ±18.05
MBP (mmHg)	86.38 ±7.29	102.02 ±5.46	110.30 ±6.58	123.70 ±15.02
HR (beats/min)	57.68 ±13.62	65.14 ±17.06	60.98 ±14.47	64.09 ±12.01
PWV (m/s)	3.42 ±01.03	3.99 ±1.09	4.77 ±01.57	5.46 ±1.50

### 3.2. Parameters Identification of the Models

For the numerical implementation of the F-MWK the definition of *Grunwald-Letnikov (GL)* given in definition 1 is used Podlubny (1999). The time validation of the proximal pressure waveforms (*P*_*p*_) was performed using two clinical hypertension real datasets. The cost function we used to calculate signal dissimilarity is the *L*_2_-norm of the difference between the two signals. It can be formally described as follows:


(12) $$\begin{array}{r} \underset{\Theta }{\text{Minimize}}||P_{p}-{\hat{P}}_{p}\left(\Theta \right)|{|}_{2}, \end{array}$$

The optimizer algorithm uses the measured aortic root flow rate as an input and compute the required model parameters, $\Theta =\left\{C_{p}^{\alpha },L,C_{d}^{\beta },R_{p},P_{v},\alpha ,\beta \right\}$, which minimize the pressure root mean square error (RMSE), i.e., the difference between measured and calculated aortic root pressure as:


(13) $$\begin{array}{r} \text{RMSE}=\sqrt{\frac{1}{N}\overset{n}{\underset{i=1}{\sum }}{\left(P_{p_{\left[i\right]}}-{\hat{P}}_{p_{\left[i\right]}}\right)}^{2}}. \end{array}$$

where *N* denotes the number of samples per *P*_*p*_ pressure signal. The estimation process was based on a non-linear least square minimization routine applying the well-known MATLAB−R2020b, function fmincon. The estimate of Θ is $\hat{\Theta }$ were found *via* the solution of the inverse problem of the estimated proximal blood pressure ($\hat{P_{p}}$) and the real one (*P*_*p*_). Initialized by Θ_0_ and using a nonlinear programming solver, the inverse algorithm iteratively predicts the set of parameters $\hat{\Theta }$ which minimizes the objective function. In this process, we constrained all the parameters to be positive to guarantee physical properties (Lower_bounds = [0], Upper_bounds = [∞] and. Once a tolerance of error was reached, the convergence of the method is confirmed, the *fmincon* function exits and yields an output of the optimal set of model parameters estimates ${\hat{\Theta }}^{*}$.

In addition, to evaluate the performance of the estimation, we calculate the relative error, R.E.(%) and the correlation coefficient, ρ defined as:


(14) $$\left\{ \begin{array}{l} \text{R}.\text{E}.(\%)=\frac{||P_{p}-{\hat{P}}_{p}|{|}_{2}}{||P_{p}|{|}_{2}}\times 100\%\\ \rho =\frac{\sum_{i=1}^{n}\left(P_{p}-\bar{P_{p}}\right)(\hat{P_{p}}-\bar{\hat{P_{p}}})}{\sqrt{\sum_{i=1}^{n}{\left(P_{p}-\bar{P_{p}}\right)}^{2}}\sqrt{\sum_{i=1}^{n}{\left(\hat{P_{p}}-\bar{\hat{P_{p}}}\right)}^{2}}}, \end{array}\right.$$

where $\bar{.}$ represents the average operator.

### 3.3. Sensitivity Analysis for the Apparent Compliance

In order to study how the variation in the apparent arterial compliance modulus and phase is associated with the variations of the different input parameters factors, a global sensitivity analysis based on *variance method* has been performed. Variance-Based Sensitivity Analysis (VBSA) is a valuable step in the model calibration process, estimating the model parameters. In fact, it provides a relevant insight on how changes in the estimates of the parameters (the inputs of the model) map into variations of the performance metric that evaluates the model fit. A detailed review with practical workflow about the sensitivity analysis literature can be found in (Pianosi et al., 2015, 2016; Wagener and Pianosi, 2019).

In this study, we evaluate the VBSA of the fractional-order arterial compliance using *First-order indices* known also as “*main effect”* and the *total-order indices* so-called “*total effect.”* The “*main effect”* indices measure the direct contribution of the output variation from individual input factor or, equivalently, the expected reduction in output variance that can be obtained when fixing a specific input (Pianosi et al., 2016). The *First-order indices* is defined as:


(15) $$\begin{array}{r} \text{VBS}{\text{A}}_{\text{F}}=\frac{V_{x\sim i}\left[E_{x\sim i}\left(y|x_{i}\right)\right]}{V\left(y\right)}=\frac{V\left(y\right)-\left[E_{x\sim i}\left(y|x_{i}\right)\right]}{V\left(y\right)} \end{array}$$

where *E* denotes expected value, *V* denotes the variance, *x* denotes the input, *y* denotes the output, and *x*~*i* denotes “all input factors but the *i*th.”


(16) $$\begin{array}{r} \text{VBS}{\text{A}}_{\text{T}}=\frac{E_{x\sim i}\left[V_{x_{i}}\left(y|x\sim i\right)\right]}{V\left(y\right)}=1-\frac{V_{x\sim i}\left[E_{x_{i}}\left(y|x\sim i\right)\right]}{V\left(y\right)} \end{array}$$

## 4. Results

In this section, we show the results of applying the proposed model for both subjects of the clinical dataset 1 and the groups of the clinical dataset 2 as described in Table 1. To fully identify the proposed fractional-order model the parameters and the fractional differentiation orders have to be estimated using the measured flow and pressure waveforms. In addition, we present the result of applying the variance-based global sensitivity analysis technique to the proposed arterial representation and the analysis of ranking the lumped parameters in order of importance based on VBSA_F_ and VBSA_T_.

### 4.1. Model Calibration

The list of the optimized F-MWK parameters and their numerical values are shown in Table 2. For clinical dataset 2, the parameter estimates' mean value and standard deviation (mean ± SD) are presented per group. The optimized parameters for each subject are then used to reconstruct the aortic proximal blood pressure waveform (*P*_*p*_). The pressure root mean square error RMSE and the percentage relative error [*R*.*E*. (%)] along with the correlation coefficient (ρ) between the real aortic pressures and the reconstructed ones are also listen in Table 2 as performances. In the following we present the detailed analysis of the proposed fractional-order model and its validation against each in-human hypertension clinical datasets.

**Table 2:** The optimized parameters and corresponding RMSE, *R*.*E*.(%) and ρ of the fractional-order modified Windkessel model for each subject of the hypertension clinical dataset 1 and different groups of the clinical hypertension dataset 2.

		**Parameters estimates**							**Performances**		
		** ${\text{C}}_{p}^{\alpha }$ **	***L***	** $C_{d}^{\beta }$ **	***R*_*p*_**	***P*_*v*_**	**α**	**β**	**RMSE**	***R.E. (%)***	**ρ**
Dataset 1	Pre-hypertension	1.48	0.052	0.37	0.81	10.69	1.001	0.771	2.23	2.26	0.99
	Mild-hypertension	0.58	0.010	0.55	2.52	15	1.023	0.540	2.27	1.90	0.99
	Severe-hypertension	0.54	0.087	0.34	3.64	15	1.000	0.452	3.025	2.19	0.99
Dataset 2	Group 1	2.77 ± 1.50	0.023 ± 0.020	2.39 ± 1.28	3.10 ± 1.37	13.90 ± 4.11	1.114 ± 0.03	0.466 ± 0.10	1.48 ± 0.50	1.70 ± 0.59	0.99 ± 0.005
	Group 2	2.00 ± 1.23	0.027 ± 0.015	1.68 ± 1.01	2.52 ± 1.05	11.85 ± 2.94	1.111 ± 0.03	0.498 ± 0.18	1.66 ± 0.45	1.62 ± 0.44	0.99 ± 0.005
	Group 3	1.61 ± 1.24	0.038 ± 0.022	1.34 ± 0.85	2.95 ± 1.24	12.37 ± 3.43	1.112 ± 0.04	0.501 ± 0.12	1.90 ± 0.50	1.70 ± 0.45	0.99 ± 0.003
	Group 4	1.13 ± 0.58	0.036 ± 0.018	0.98 ± 0.47	2.09 ± 1.12	11.88 ± 3.26	1.130 ± 0.05	0.572 ± 0.19	2.34 ± 0.62	1.87 ± 0.44	0.99 ± 0.002

*For each group the numerical mean value and standard deviation (mean ± SD) of the estimates and performance are presented*.

#### 4.1.1. Human Hypertension Dataset 1

In order to validate and check the ability of the F-MWK in reconstructing the proximal blood pressure at various levels of hypertension conditions, namely the Pre-hypertension, Mild-hypertension, and severe hypertension in this part, we use human data from the hypertensive study, (Nichols et al., 1993). The lumped model parameters are identified using the aortic blood flow (*Q*) as an input. The reconstructed proximal blood pressures after applying F-MWK using the identified parameters along with the experimental *in-vivo* waveform are shown in Figures 2A,C,E represent the model input aortic-valve blood flow rate (*Q*) for each hypertension condition level and Figures 2B,D,F represent the pressure waveform validation results. Based on this result, it is clear that the proposed fractional-order model captured the main features of the proximal aortic pressure waveform, including the maximum value (peak systolic value) and the dicrotic notch. The model conforms better in detecting these features in the cases of Mild-hypertension and Severe-hypertensive level, where the percentage relative errors were 1.9 and 2.19%, respectively. The correlation coefficients for all the studied cases are around 0.99, which confirms the model's capability to catch the explicit details of the arterial blood pressure morphology.

In addition, the model presents a better performance in terms of RMSE than the models presented in Aboelkassem and Virag (2019) namely the hybrid Windkessl-Womersley (WK-W) model. for instance, the RMSE value was around 3.02 in the case of F-MWK; however, it is equal to 4.12 in the case of (WK-W). It is worth noting that WK-W is a hybrid model that consists of the proximal and distal compartments similar to F-MWK; however, these two compartments are connected by a tube to represent the aorta where the blood flow is expressed by the Womersley solution of the Navier-Stokes equations. Accordingly, the fractional-order framework can reproduce an accurate performance similarly to more complex systems. By interpreting the numerical values of the proximal and distal pseudocapacitances ($C_{p}^{\alpha }$, $C_{d}^{\beta }$) and the corresponding fractional differentiation-orders (α, β), we notice a clear decrease of these parameters from Pre-hypertension level to the Sever-hypertension level.

On one side, this result demonstrates the fractional-order behavior within the distal arterial network. In fact, β is less than the integer-order and takes values between 0 and 1. In addition, as the level of hypertension increases, the fractional differentiation order decreases. Furthermore, from equation (7), it is obvious that as β alters from 1 to 0, the FOC's resistive part increases. Accordingly, the results of the identified fractional-order parameters are consistent with the clinical investigations, which have revealed that the vascular remodeling in resistive arteries is strongly associated with the progression and severity of hypertension's disease.

#### 4.1.2. Human Hypertension Dataset 2

To further validate and interpret the efficiency of the developed model, we explore the second clinical dataset that consists of 158 human subjects examined as hypertensive patients. A portion of the studied population is under treatment, and their high blood pressures were controlled and regulated to be within the normal values. The main objective of investigating this type of data is to keep the generality of the proposed model and demonstrate that this model can be employed in different physiological conditions. In fact, the dataset presents patients of different ages, gender, weight, and hemodynamic characteristics as listen in Table 1.

**Figure 3:**
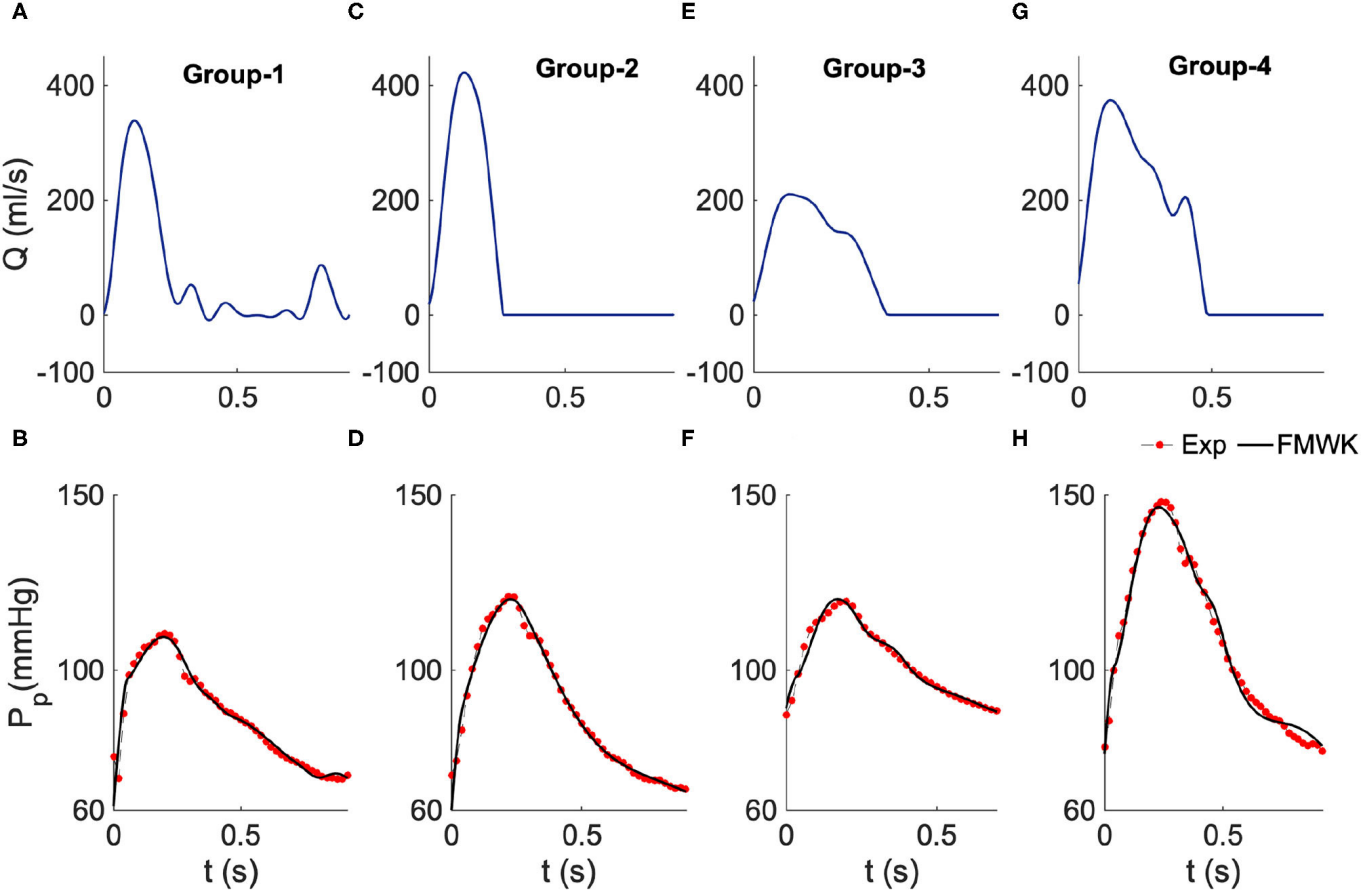
Estimated proximal blood pressure using the proposed fractional-order modified Windkessel model along with the experimental *in-vivo* human-hypertension for samples from the different groups of the dataset 2. **(A,C,E,G)** Represent the model input aortic-valve blood flow rate for each group's sample study, Group 1, Group 2, Group 3 and Group 4, respectively. **(B,D,F,H)** Represent the pressure waveform validation results.

Basically, we divided this data into four classes based on the peak systolic blood pressure and diastolic values, SBP and DBP, respectively. The thresholds of SBP and DBP were set in a manner that: Group 1 consists of patients with regulated blood pressure levels that can be considered as a normotensive subset, Group 2 consists of patients with a bit high SBP and DBP, which some studies consider it as Pre-hypertension stage, Group 3 consist of patients with high-level blood pressure values which is indeed thought as hypertension subset, and Group 4 with the highest SBP and DBP is supposed to present the severe-hypertensive samples. Figure 3 summarizes the result of the proximal aortic blood pressure reconstruction of a representative patient from each group. The proposed model can capture all the waveforms details, including the dicrotic notch and the peak systolic value. It is worth noting that the selected patients present different aortic input blood flow profiles.

As shown in Table 2 the values of the performance indexes, namely RMSE, *R*.*E*.(%) and ρ, indicate that the proposed model was able to reconstruct the proximal blood pressure using the optimized lumped parameters. The RMSE values do not exceed 3 in all cases, and the smallest value is around 1.48 ± 0.50 for group 1, and the largest one is 2.34 ± 0.62 for group 4. Also the smallest *R*.*E*. is around 1.62% ± 0.44% obtained for group 2 and the largest one is 1.70% ± 0.59% for group 1. The correlation coefficient is around 0.99 for all the groups.

By checking the optimized values of the fractional-order parameter, namely the pseudocapacitance ($C_{p}^{\alpha }$, $C_{p}^{\beta }$) and their corresponding fractional differentiation orders (α, β), we noticed that $C_{p}^{\alpha }$ and $C_{p}^{\beta }$ decrease from group 1, which considered representative of the normotensive population to group 4 that corresponds to the subset with severe-hypertensive level based on the value of SBP and DBP. However, α and β slightly increase from groups 1 to 4. This result is different from the one found with the clinical subset 1. This can be explained by the fact that the presented model is not globally identifiable. It is very challenging to find unique values for the pairs ($C_{p}^{\alpha }$, α) and $C_{d}^{\beta }$, β. Accordingly, it is more pertinent to evaluate the complex and frequency-dependent compliance *C*_*C*_ that conveys the relationship between the fractional differentiation order and the pseudocapacitance *via* the expression (9). The following section focuses on the evaluation and analysis of *C*_*C*_*h*__ at the cardiac frequency for both proximal and distal compliances.

### 4.2. Variance Based Sensitivity Analysis

Generally, to simulate the proximal blood pressure waveform, we feed F-MWK with the identified set of the values of the lumped parameters, which can be regarded as a scalar input of the proposed model. In order to test and understand the effect of varying one of the inputs at a time on the output signal dynamic and morphology and study the interactive effect between the inputs and the output, we conduct a variance-based sensitivity analysis (VBSA) as explained in the method section.

Two indices based on VBSA were evaluated in this study: the first-order index (VBSA_F_), which reflects the main effect contribution of each input factor to the variance of the output and ranks the importance of this input; the second one is the total effect index (VBSA_T_) which accounts for the total contribution to the output variation due to factor input evaluated by the first-order effect in addition to all higher-order effects due to interactions. In this study both indices were evaluated at each sample of the proximal aortic blood pressure waveform for the three hypertensive subject of the clinical dataset 1.

**Figure 4:**
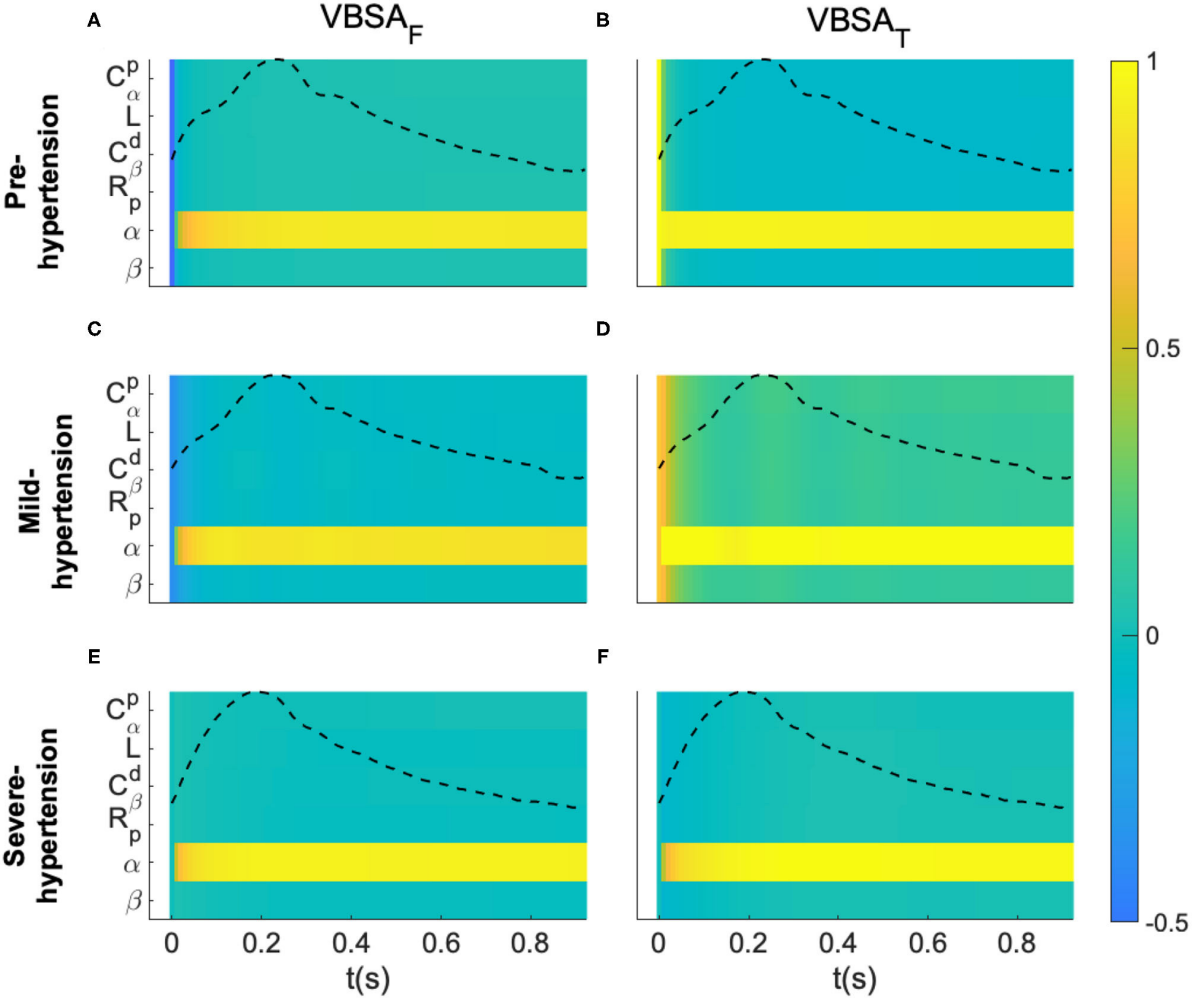
Variance-based sensitivity analysis of the proposed fractional-order model. **(A,C,E)** Represent the first-order indices (VBSA_F_) of the model's parameters for a Pre-hypertensive, Mild-hypertensive and Severe-hypertensive subject, respectively. **(B,D,F)** Represent the total-order indices (VBSA_T_) of the model's parameters for a Pre-hypertensive, Mild-hypertensive and Severe-hypertensive subject, respectively. The variance based sensitivity indices were evaluated for each sample of the aortic proximal blood pressure over one cardiac cycle.

We consider a normal distribution variation of each input parameter $X\sim N\left(\mu ,\sigma^{2}\right)$ where *X* corresponds to the input, μ is the mean value of the distribution that is equal to the optimized value of the parameter shown in Table 2. (Dataset 1) and σ denotes the standard deviation that is taken to be 15% of the value of μ. Figures 4A,C,E show the evaluated VBSA_F_(t) for the Pre-hypertension, Mild-hypertensive and Severe-hypertensive patients, respectively. Figures 4B,D,F displays the evaluated VBSA_T_(t) for the Pre-hypertension, Mild-hypertensive and Severe-hypertensive patients, respectively. For all cases the indices were computed over one cardiac cycle.

For visualization purposes of each subject, all the parameters are listed in *y-axis*, whereas the *x-axis* represents the time samples. In addition, the normalized blood pressure waveform was plotted in the same plot. It is very clear from these results that F-MWK is very sensitive to the variation of the fractional differentiation order (α) over the whole cardiac cycle for all the hypertension levels. The rest of the parameters are less influential on the output dynamic, though their effect varies from one hypertension level to another. In fact, this effect is more considerable in the Mild-hypertensive case, as shown in subplot D. Generally, the difference between VBSA_T_(t) and VBSA_F_(t) measures how much the parameter is involved in the interaction with other input factors. Accordingly, the parameters are very affected by any interaction between the input factor in the case of Mild-hypertension. Based on these observations, the fractional differentiation orders might have central control in the variation of the aortic blood pressure. Accordingly, this parameter might play an important position as a bio-marker assessing the transition between viscosity and elasticity, a potential arterial stiffness index.

## 5. Discussion

The fractional-order capacitor represented by its pseudocapacitance and the fractional differentiation order can be an inherent component in F-MWK by lumping the complex and frequency-dependent behavior of the vascular compliance as well as characterizing the hemodynamic. The sensitivity analysis and the model calibration show that the fractional-order pairs may entail valuable structural and functional physiological insight. Based on the validation results using clinical dataset 1 a clear, direct correlation between the fractional differentiation orders and the level of hypertension was found in agreement with the clinical analysis. Indeed, as the level of hypertension increases, a decrease in the numerical values of the fractional differentiation orders (α and β), as well as the pseudocapacitances ($C_{p}^{\alpha }$ and $C_{d}^{\beta }$), was reflected.

The concurrent decrease of the fractional-order parameters of FOC yields to the predominance of the resistive (dissipative) part on account of the capacitive (storage) part in this element. This fact is a potential representative of vascular remodeling associated with the severity of hypertension. Analyzing the fractional differentiation orders independently from the pseudo-capacitance might lead to misinterpretation. In fact, based on expression (5), these two parameters might have a compensatory inter dynamic mechanism. Accordingly, it is more appropriate to evaluate the complex fractional-order compliance that relates both parameters *via* expression (7) at the heart pulsation (ω_*h*_), reflecting the so-called true arterial compliance. It is defined as follow:


(17) $$\begin{array}{r} C_{C_{h}}=C_{C}\left(\omega_{h}\right)=C_{app}^{\alpha }{\left(j\omega_{h}\right)}^{\alpha -1}. \end{array}$$

**Figure 5:**
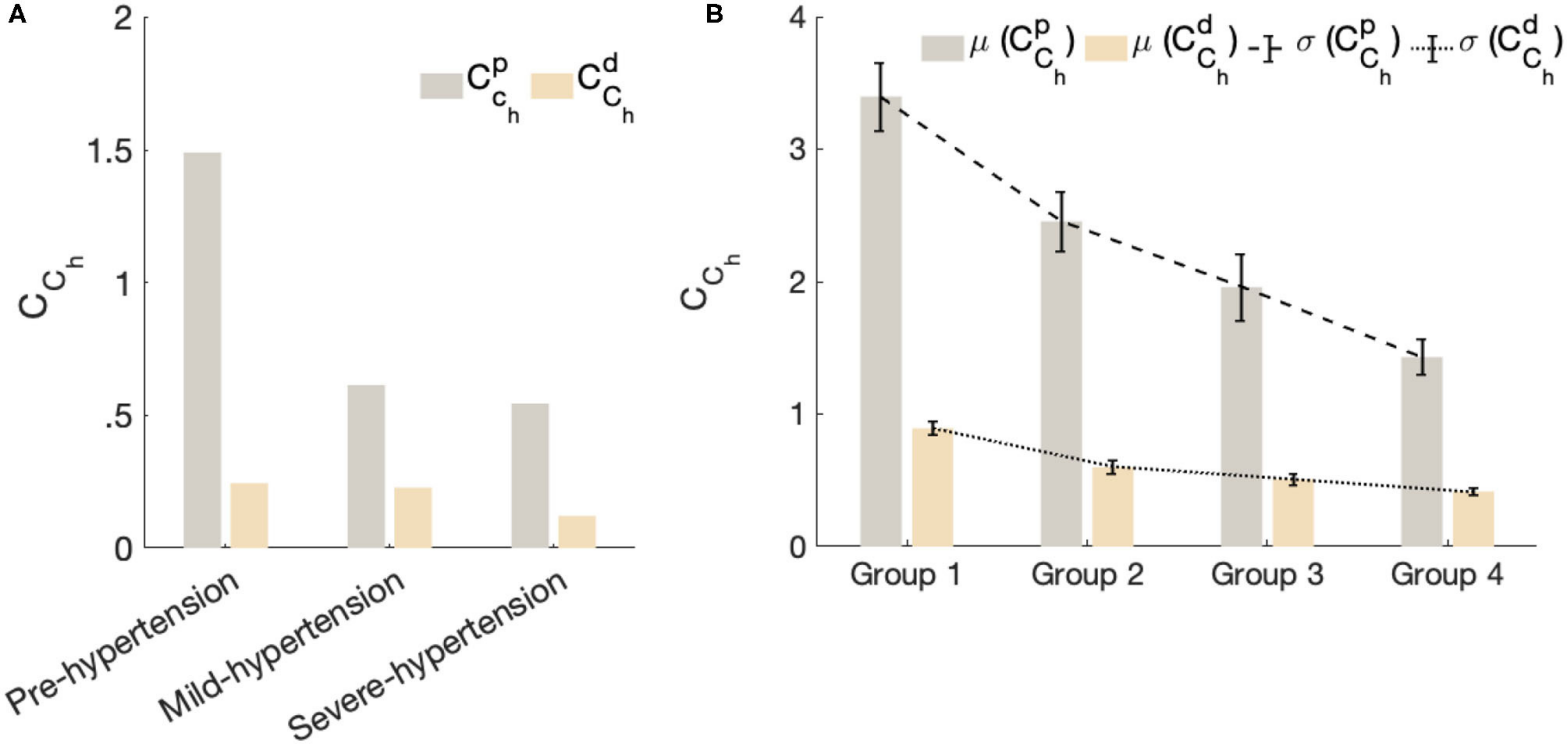
The fractional-order compliance evaluated at the heart pulsation. **(A)** Represents bar plot of the proximal ($C_{C_{h}}^{p}$) and distal ($C_{C_{h}}^{d}$) fractional order compliance for the in-human hypertensive subjects of dataset 1. **(B)** Represents error bars plot of the proximal ($C_{C_{h}}^{p}$) and distal ($C_{C_{h}}^{d}$) fractional order compliance for the in-human hypertensive groups of dataset 2. Each bar in this plot represent the mean value of the evaluated fractional-order compliances per group and the error corresponds to the standard error of the mean per group.

Figure 5 shows the fractional-order compliance evaluated at the heart pulsation: Figure 5A represents the bar plot of the proximal ($C_{C_{h}}^{p}$) and distal ($C_{C_{h}}^{d}$) fractional-order compliance for the in-human hypertensive subjects of dataset 1; Figure 5B represents the error bars plot of the proximal ($C_{C_{h}}^{p}$) and distal ($C_{C_{h}}^{d}$) fractional-order compliance for the in-human hypertensive groups of dataset 2. The error in this plot corresponds to the standard error of the mean. From this figure, in all cases, $C_{C_{h}}^{p}$, which characterizes the large elastic arteries, is larger than $C_{C_{h}}^{d}$, which characterizes the small resistive vessels. In addition for both datasets, as the hypertensive increases both compliances decreases. From Figure 5B, the gradient from group 1 representing a normotensive population to group 2 (Pre-hypertensive) is more acute than the slopes between groups 2 and 3 and groups 3 and 4, which are approximately equal. *C*_*C*_*h*__ is substantially decreased from normal blood pulse pressure to the higher one.

**Figure 6:**
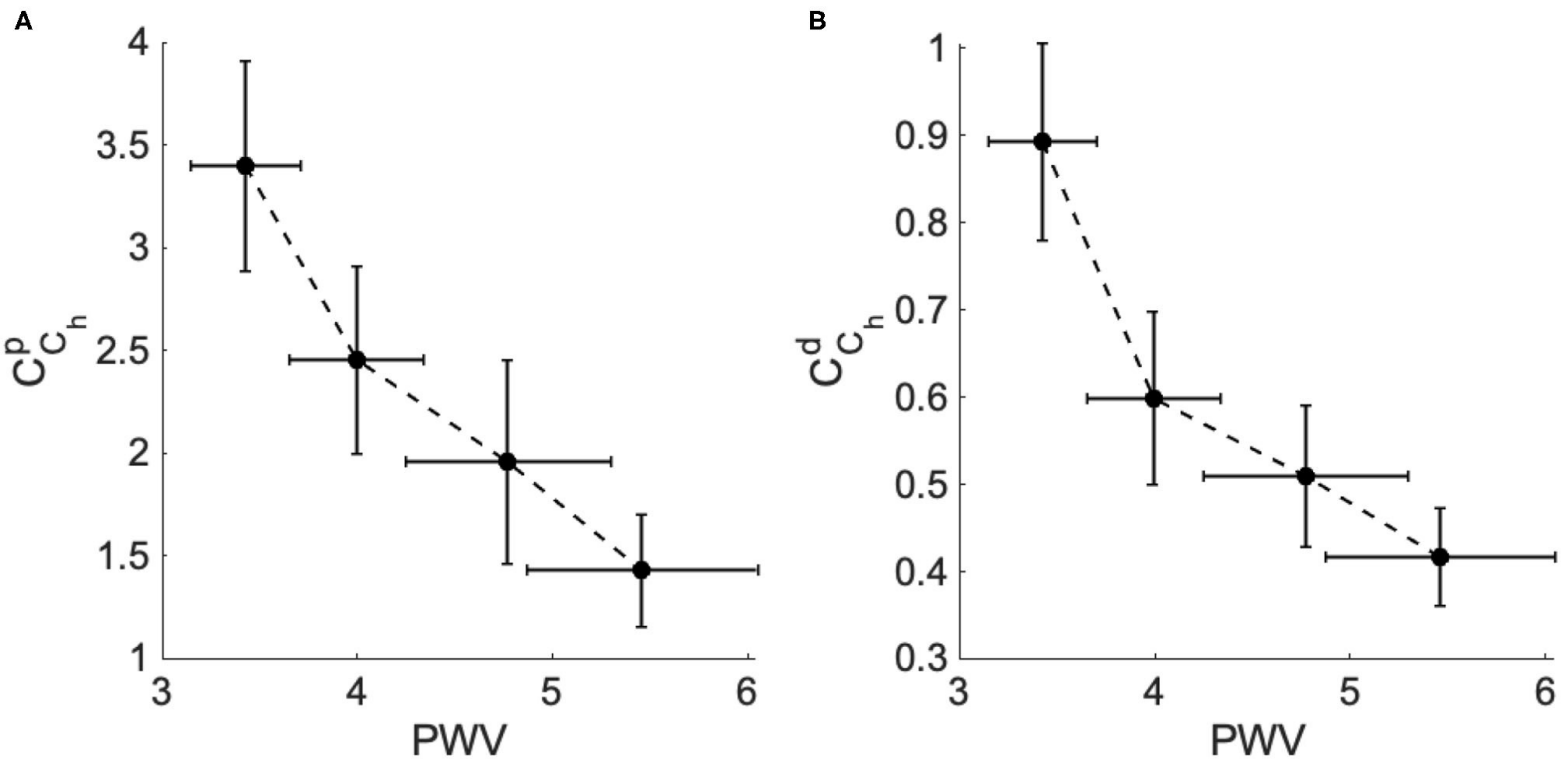
The fractional-order compliance evaluated at the heart pulsation (*C*_*C*_*h*__) vs. the arterial pulse wave velocity (PWV) for the different hypertensive groups of dataset 2. **(A)** PWV vs. the proximal fractional-order compliance ($C_{C_{h}}^{p}$) and **(B)** PWV vs. the distal fractional-order compliance ($C_{C_{h}}^{d}$).

This result is very important and demonstrates the reliability of *C*_*C*_*h*__ in characterizing stiffness as an important risk factor for the progression of high blood pressure. As the clinical dataset 2 provides the aortic pulse wave velocity (PWV) of each patient, in Figure 6. we plotted the mean values for both $C_{C_{h}}^{P}$ and $C_{C_{h}}^{d}$ for each group vs. the mean values of PWV for each group. The result shows the strong negative correlation between the PWV and *C*_*C*_*h*__ which is in agreement with the clinical standard that shows that PWV values substantially increase with the hypertensive level as a consequence of arterial stiffness.

The correlation with PWV (a well-established biomarker of along with the level of hypertensive reveals the potential of the fractional-order parameters to improve our understanding of the structural development of vascular remodeling due to hypertension. Indeed, fractional-order parameters are considered a prospective new tool in capturing irregular vascular changes. Stress-relaxation-based viscoelastic experiments on arterial segment have reported that the fractional differentiation order parameter could be associated with vascular smooth muscles cells (VSMCs) activity, contributing to the viscoelasticity modulation in the vessels (Craiem and Armentano, 2007; Craiem et al., 2008).

In arteries, VSMCs induce the stretching of collagenous fibers, and vascular activation can modify the local viscoelastic response of the arterial wall (Armentano et al., 2006). Vascular smooth muscle cells (VSMCs) represent an important part of blood vessels and are placed in the medium portion of the arterial vessel, known as tunica media. They are located circularly around the vascular lumen and other vascular layers. VSMCs play a crucial role in the remodeling processes of the vascular wall due to certain diseases.

In hypertension, the vascular remodeling induces different changes in the VSMCs of large and resistive small arteries: with regards to larger vessels, VSMCs experience hypertrophy remodeling, an expansion in the cellular vessel material, which results in an enlarged intima-media thickness, raised vascular stiffness, and so high blood pressure (increased pulse pressure). In the small arteries, the vascular remodeling manifests as a eutrophic phenomenon, which results in an increase in wall thickness and a reduction in lumen diameter. It can represent hypertrophic reconstruction as well.

Several clinical studies in-patient and experimental researches have revealed the marked correlation between vascular remodeling and the pathophysiology of hypertension. Low dimensional models (lumped parameters models) are quite limited in the vascular remodeling structural analysis contest, which requires an extremely complex interplay of deeply coupled multi-scale and multi-physics mechanisms. The fractional-order framework offers much promise in understanding key physiological mechanisms while reducing the order of complexity. Due to the extra fractional-order parameters, more flexibility is added to capture structural artery characteristics.

## 6. Model Development and Future Applications

Fractional order modeling (FOM) approach can be viewed as a natural generalized of the well-known blood flow arterial Windkessel model. The proposed model can be easily integrated within a closed-loop whole heart lumped parameter model representation for better understanding of the blood flow dynamics in the cardiovascular system. In this part, we list a couple of applications where the present model can be useful and is expected to perform well.

The first application is related to using the present model as a surrogate measure of arterial stiffness prediction. For example, it is commonly known that probing arterial stiffness at the arterial locations provides valuable information about the physiological state of the cardiovascular system. An increase in arterial stiffness plays a critical role in the pathogenesis of cardiovascular disorders and is identified as a major risk factor for many cardiovascular pathologies such as hypertension and coronary heart diseases. Accordingly, the present model can be used within a larger integrative computational platform for assessing arterial stiffening at different distal locations. Hence, it can be used for better understanding arterial blood flow, predicting, and diagnosing many stiffness related cardiovascular diseases.

The second application is related to the use of the pulsed wave velocity (PWV) as a standard method for assessing arterial stiffness. The PWV is functionally assessed by evaluating the time it takes for the pressure waveform to travel two known arterial sites. Despite its wider adoption in the clinical routine, the measurement process of PWV is considered a demanding task for both clinicians and patients. Since the present model have the capability of estimating the PWV. Therefore, it can be used toward developing a full scale non-invasive and easy-to-use computational tool that can overcome the challenges of the classical assessment stiffness process and hence it can be used to improve the quality of patient care.

In summary, the present model is an initial step toward developing an integrative non-invasive surrogate markers of local and global arterial stiffness. The marker is based on the fractional differentiation order that controls the transition between the resistive and capacitive parts of the fractional-order element and, by analogy, represents the viscoelastic properties of the vasculatures.

## 7. Conclusion

A novel fractional-order lumped model of the arterial system is proposed to study hypertension. The model has shown the feasibility of characterizing the proximal and distal arterial compliances using fractional-order capacitors. The *in-vivo* human validation demonstrates the ability of the proposed model in reconstructing the central blood pressure and capturing specific details of different waveforms morphology. The variation of the complex and frequency-dependent apparent arterial compliance evaluated at the heart pulsation vs. different hypertensive levels shows consistency with the clinical observations. Moreover, the correlation with the pulse wave velocity (a well-established biomarker of arterial stiffness) demonstrates the capabilities of this model, namely the fractional-order parameters. The model results are expected to improve our understanding of the structural and functional characteristics of the resulting vascular remodeling of large and small arteries in different hypertensive conditions.

## Data Availability Statement

The original contributions presented in the study are included in the article/supplementary material, further inquiries can be directed to the corresponding author/s.

## Author Contributions

MB: study conception and design. MB and YA: data collection. MB, YA, and T-ML-K: analysis and interpretation of results and draft manuscript preparation. All authors reviewed the results and approved the final version of the manuscript.

## Funding

Research reported in this publication has been supported by the King Abdullah University of Science and Technology (KAUST).

## Conflict of Interest

The authors declare that the research was conducted in the absence of any commercial or financial relationships that could be construed as a potential conflict of interest.

## Publisher's Note

All claims expressed in this article are solely those of the authors and do not necessarily represent those of their affiliated organizations, or those of the publisher, the editors and the reviewers. Any product that may be evaluated in this article, or claim that may be made by its manufacturer, is not guaranteed or endorsed by the publisher.
